# Botulinum neurotoxin (BoNT) treatment in functional movement disorders: long-term follow-up

**DOI:** 10.1136/jnnp-2020-323684

**Published:** 2020-07-13

**Authors:** Yasmine EM Dreissen, Franka Lambert, Joke M Dijk, Johannes HTM Koelman, Marina AJ Tijssen

**Affiliations:** 1 Neurology, Amsterdam University Medical Centres, Amsterdam, Noord-Holland, The Netherlands; 2 Clinical Neurophysiology, Amsterdam University Medical Center (AMC), University of Amsterdam, Amsterdam, Noord-Holland, The Netherlands; 3 Neurology, University Medical Centre Groningen, Groningen, The Netherlands

**Keywords:** movement disorders, functional neurological disorder, botulinum toxin

## Introduction

We recently reported on a randomised controlled trial (RCT) assessing the effect of botulinum neurotoxin (BoNT) in 48 patients with chronic (>1 year) jerky and tremulous functional movement disorders (FMD).^1^ The RCT showed an improvement of motor symptoms in both treatment arms (16/25, 64% BoNT vs 13/23, 57% placebo). The proportion of improved patients increased to 81% (35/43) at the end of the open-label phase. Despite symptom improvement, there was no change in quality of life and disability. In the present study, our aim was to assess the long-term outcome of this study population.

## Methods

### Population and study design

Included patients were aged between 18 and 80 years with disabling functional jerky or tremulous movement disorders with a minimum symptom duration of 1 year.^1^

### Procedures

All 48 patients who participated in the BoNT RCT were approached via letter or email. Patients were interviewed by telephone (FL) and self-assessment questionnaires were sent to patients’ homes. We compared the outcome measures of the current study with baseline (start RCT) and the end of the open-label phase.

### Outcome measures

A selection of previously used outcome measures was used.^1^ Self-rated motor improvement (Clinical Global ImpressionImprovement Scale (CGI-I)) and motor severity (Clinical Global Impression-Severity-Scale (CGI-S)), disease burden (VAS scale), physical functioning (Short-Form-36 (Sf-36)), depressive symptoms (Beck Depression Inventory (BDI)) and anxiety symptoms (Beck Anxiety Inventory (BAI)) were evaluated. Patients were questioned about their current employment status, BoNT (or other) treatment and new functional neurologic symptoms (FNSs).

### Statistical analysis

Demographic characteristics were analysed using descriptive statistics. Patients were categorised based on CGI-I between baseline and the end of the follow-up into three groups. The first group, or ‘improved’ group (group 1), the second group, or ‘no change’ group (group 2), and the third group, or ‘worse’ group (group 3).

The CGI-S, VAS, BAI, BDI and SF-36 were compared between the different time points using a Wilcoxon singed-rank test. Additionally, symptom improvement (CGI-I) and severity (CGI-S) were compared between patients who did and did not still receive BoNT treatment using Fisher’s exact test or Mann–Whitney U test. The threshold for statistical significance was set at a two-sided p<0.05. All analyses were performed using IBM SPSS Statistics V.24.

## Results

Of the 48 patients who were approached, 46 patients agreed to participate. Six patients completed the telephone interview, but did not return the questionnaires and nine only agreed to answer a subset of questions. The follow-up duration varied between 3 and 7 years (median years (IQR 3–6)). In total, CGI-I scores were available in 42 patients. The other outcome measures were available in n=46 patients.

The median age of patients at follow-up was 58 years (IQR 42–65), 54% was male. The median symptom duration was 9 years (IQR 7–18). Since the end of the open-label phase of the BoNT trial, 7 of 37 patients reported new functional symptoms. Of the 37 patients who completed the telephone interview, 2 had started working and 3 had quit work since baseline. Thirteen of these 37 patients reported that they received one or more new therapies targeting FMD. Ten out of these 13 patients reported symptom improvement attributed to these therapies, 2 remained unchanged and 1 worsened.

**Figure 1:**
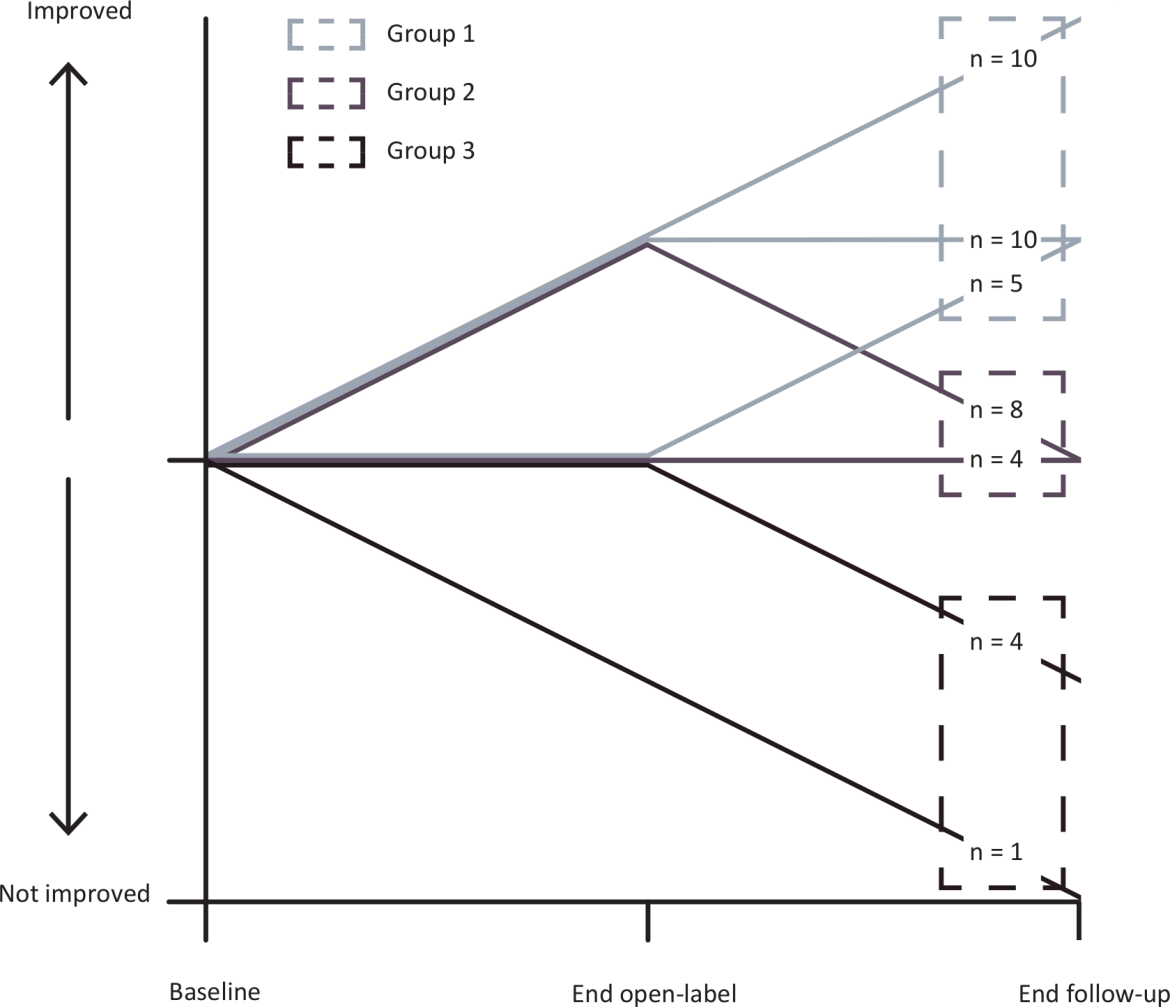
Disease course in the three subgroups based on CGI-I scores at baseline, end-open label study and the end of the follow-up.

The disease course based on the CGI-I between the three time points is illustrated in figure 1. For the entire group (n=46), motor severity (CGI-S) improved between baseline and long-term follow-up (median 5 vs 4, p=0.00). Disease burden (VAS) improved significantly between baseline and the end of the open-label phase (median 15 points; p=0.02) but returned to baseline levels at the long-term follow-up (p=1.00). Depressive symptoms improved slightly between baseline and end of open-label (two points; p=0.02) but returned to baseline levels during the long-term follow-up (for details, see online supplementary data file 1).

10.1136/jnnp-2020-323684.supp1 Supplementary data

### BoNT treatment

Of the 17 patients who continued BoNT treatment after the open-label phase, 10 still received BoNT at the long-term follow-up. Eight patients were stable or improved and two patients worsened. No differences in symptom improvement or severity were found between patients with and without BoNT treatment.

## Discussion

This study showed motor improvement at the end of long-term follow-up in the majority of patients (25/46), although the proportion slightly diminished compared with the end of the open-label study (35/43). Still, this is more favourable than what one would expect based on current literature. Recently, Gelauff *et al* 2 reported on the long-term (average 14 years) outcome of a large cohort (n=107) of patients with motor FNS in which 49% had persistent or worsening of symptoms. This is in line with a systematic review on the prognosis of FMD.^3^ The natural course however of FMD is unknown. Because the number of patients in this cohort currently receiving BoNT is small, it is not possible to assess whether botulinum treatment has an additive effect. Also, 13 of 46 patients received other therapies for FMD, which they largely considered effective. Regression to the mean, the natural disease course and placebo effects may play a role.

Similar to our previous study, the improvement in motor symptoms did not translate into improvement of physical functioning or disease burden. It remains a matter of speculation why there seems such a poor relation between motor and non-motor symptoms. A case–control study comparing FMD with neuromuscular disorders found that quality of life was not affected by motor symptoms.^4^ An important issue is the lack on validated and clinically relevant outcome measures in FNS,^5^ generating uncertainty in interpreting the results of the different outcome measures in this patient group.

This study has several limitations. First, the follow-up term varied in duration. We used a selection of subjective instead of objective outcome measures and data were not available in all patients.

In conclusion, this long-term follow-up study reveals a favourable motor outcome in the majority of patients with chronic FMD. The improvement may be largely due to placebo effects. It shows though that treatment studies in this difficult patient category are important and potentially rewarding.
