# Supplementary material for: Botulinum neurotoxin (BoNT) treatment in functional movement disorders: long-term follow-up
Source: J Neurol Neurosurg Psychiatry. 2020 Jul 13;91(10):1120–1. doi: 10.1136/jnnp-2020-323684 (PMC7509513; doi:10.1136/jnnp-2020-323684)
Supplement: Supplementary data [file jnnp-2020-323684supp001.pdf]

Supplementary data file. Secondary outcome measures

	**Study Population n=46**						
	**Baseline**	**End of open label phase**	**Long-term follow-up**	**Change score (Long-term follow-up vs end of open label phase)**	**p value**	**Change Score (Long-term follow-up vs baseline)**	**p value**
**CGI-S**	5 (4;6)	4 (2;5)	4 (2;5)	0 (-1;1)	0.69	-1 (-2;0)	**0.00**
**VAS**	56 (36;77)	25 (2;62)	55 (30;76) *	15 (-13;53)*	**0.02**	1 (-23;24)*	0.97
**Physical Functioning**	56 (40;95)	65 (36;100)	65 (40;90)*	-5 (-15;9)*	0.46	0 (-10;8)*	0.57
**BDI**	8 (5;14)	6 (3;13)	8 (2;11) *	0 (-5;3)*	0.83	-2 (-6;0)*	**0.02**
**BAI**	12 (7;16)	7 (4;15)	9 (3;14)*	2 (-3;5)*	0.33	0 (-6;3)*	0.33

Table 1. Summary of the outcome measures all expressed as median and Inter Quartile ranges (IQR). CGI-S = Clinical Global Impression - Severity; VAS= Visual analogue scale; BDI=Beck depression inventory; BAI=Beck Anxiety Inventory. Physical Functioning scale of the 36-Item Short Form Health Survey. *Data of N=31 patients.
